# Learning by Numbers in the Primate Cortex

**DOI:** 10.1371/journal.pbio.0050319

**Published:** 2007-10-30

**Authors:** Rachel Jones

When a two-year-old child learns that a short vertical line can represent the number one, and that the number one represents a single item—whatever that item is—he or she has taken a great stride in the development of symbolic thinking. Although many animals can judge numerosity (for example, which of two groups of animals is larger) and can use such judgements for important decisions, such as whether to fight a smaller group or retreat from a larger one, only humans have developed the ability to use numbers as symbols to represent numerosity in a precise manner. In addition, of course, we use symbols as language more generally, both written and verbal.

So how does the human brain learn to associate inherently meaningless symbols with important, meaningful concepts such as quantities? Diester and Nieder investigated this question by teaching two macaque monkeys to associate Arabic numerals (1, 2, and so on) with the numerosity of dot displays—for example, the numeral “1” was paired with a single dot, whereas the numeral “4” was paired with four dots. The authors then recorded the activity of individual neurons in the monkeys' brains to investigate how the brain represented this association.

In humans, two parts of the brain seem to be particularly important for processing numerical information, whether symbolic or not—the prefrontal cortex (PFC) and the intraparietal sulcus (IPS). The same areas of the monkey brain have been implicated in nonsymbolic numerical processing. It has therefore been proposed that symbolic numerical abilities in humans build on the same neural systems that are used by other animals for judgements of numerosity. Diester and Nieder recorded from these two brain areas in their macaques to test whether the PFC and IPS were used by monkeys to associate visual symbols (numbers) with specific quantities.

Over several months, the monkeys learned to use a lever to indicate whether two stimuli, shown before and after a short delay, represented the same quantity—even if one of the stimuli was an Arabic numeral and the other was a group of dots. When the authors recorded from neurons in the monkeys' brains while the monkeys were performing the task, the results showed that many neurons in both the IPS and the PFC had a preference for a specific number of dots. For example, a particular PFC neuron might respond most strongly to a group of three dots, regardless of their arrangement. The neurons also showed a characteristic “tuning function,” in which their responses became weaker as the number of dots became further from the “preferred” number. So, the neuron that responded strongly to three dots would respond less strongly to two or four dots, and still less strongly to only one dot.

**Figure:**
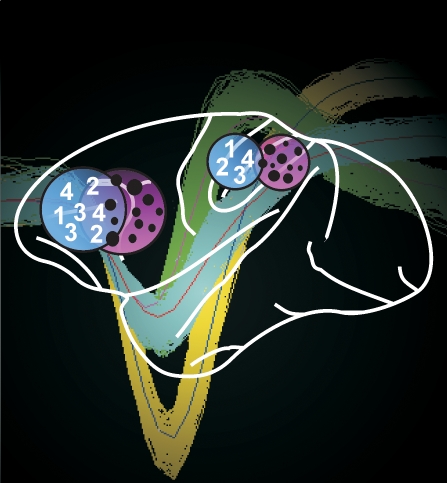
Prefrontal neurons link numerical categories to visual signs.

Many neurons in both the PFC and the IPS also showed a preference for specific Arabic numerals. However, only in the PFC did the authors find a significant number of neurons that were “tuned” to both a preferred quantity and a preferred numeral. In these neurons, the preferred numeral and quantity were correlated—that is, a PFC neuron that responded most strongly to a group of three dots was also likely to respond most strongly to the numeral “3.” Strikingly, these neurons also showed a similar tuning function, with a graded reduction in response to numerals that represented quantities progressively further from the preferred numeral.

These neurons represent the association between symbols (numerals) and numerical categories, so the authors called them “association neurons.” Their abundance in the PFC indicates that the PFC is particularly important for establishing links between symbols and categories. Interestingly, young children who are just learning about numbers show increased activity in the PFC when they are carrying out numerical activities that require associations with symbols. In older humans, who are more proficient at using numbers as symbols, there is greater activity in parietal areas such as the IPS. The authors therefore suggest that the PFC is capable of establishing symbolic associations, but that established associations are relocated to the IPS, freeing up the PFC for more demanding cognitive processes.

These results provide exciting insights into how symbolic numerical abilities might have evolved in the human brain, building on existing precursor systems that are used by other animals for numerical judgements.

